# Epitope Mapping of Neutralizing Monoclonal Antibodies to Human Interferon-γ Using Human-Bovine Interferon-γ Chimeras

**DOI:** 10.1089/jir.2016.0017

**Published:** 2016-09-01

**Authors:** Bartek Zuber, Karin Rudström, Cecilia Ehrnfelt, Niklas Ahlborg

**Affiliations:** ^1^ Swedish Orphan Biovitrum AB, Stockholm, Sweden.; ^2^ Mabtech, Nacka Strand, Sweden.; ^3^ Department of Immunology, Stockholm University, Stockholm, Sweden.

## Abstract

Our aim was to identify conformational epitopes, recognized by monoclonal antibodies (mAbs) made against human (h) interferon (IFN)-γ. Based on the mAbs' (*n* = 12) ability to simultaneously bind hIFN-γ in ELISA, 2 epitope clusters with 5 mAbs in each were defined; 2 mAbs recognized unique epitopes. Utilizing the mAbs' lack of reactivity with bovine (b) IFN-γ, epitopes were identified using 7 h/bIFN-γ chimeras where the helical regions (A-F) or the C terminus were substituted with bIFN-γ residues. Chimeras had a N-terminal peptide tag enabling the analysis of mAb recognition of chimeras in ELISA. The 2 mAb clusters mapped to region A and E, respectively; the epitopes of several mAbs also involved additional regions. MAbs in cluster A neutralized, to various degrees, IFN-γ-mediated activation of human cells, in line with the involvement of region A in the IFN-γ receptor interaction. MAbs mapping to region E displayed a stronger neutralizing capacity although this region has not been directly implicated in the receptor interaction. The results corroborate earlier studies and provide a detailed picture of the link between the epitope specificity and neutralizing capacity of mAbs. They further demonstrate the general use of peptide-tagged chimeric proteins as a powerful and straightforward method for efficient mapping of conformational epitopes.

## Introduction

Human interferon (hIFN)-γ is predominantly produced by T cells and natural killer (NK) cells, activated by immune and inflammatory stimuli, and promotes both protective innate and adaptive immune responses. It is, however, also involved in various immunopathological conditions and aberrant levels of IFN-γ are associated with a number of autoinflammatory and autoimmune diseases (Jager and others 2010; Reinhardt and others 2015). Neutralizing antibodies to hIFN-γ are therefore interesting as potential therapeutic reagents (Reinisch and others 2010; Hatterer and others 2012).

**FIG. 1.**
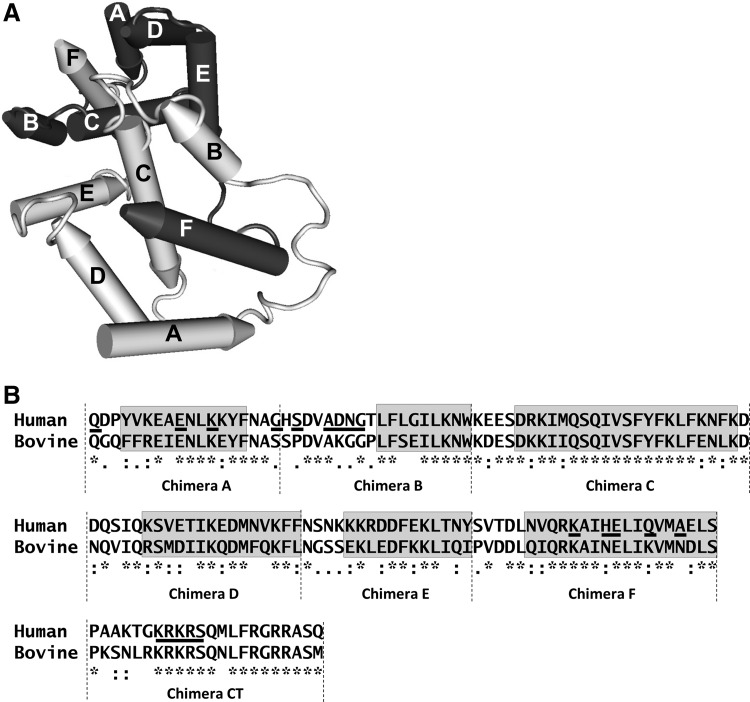
Structure of IFN-γ and human-bovine IFN-γ chimeras. **(A)** Schematic drawing of the IFN-γ homodimer with helical regions shown as cylinders interconnected by nonhelical sequences shown as thin tubes. The respective monomers are indicated by dark and light *gray* with the pointed part of each helix pointing toward the C terminus. The figure was drawn using the program CN3D (www.ncbi.nlm.nih.gov/Structure/CN3D/cn3d.shtml) based on X-ray chrystallography data for bIFN-γ (Randal and Kossiakoff, 2000), which is very similar to hIFN-γ. **(B)** The aligned amino acid sequences of hIFN-γ and bIFN-γ are shown with helical regions *boxed* and denoted A through F. Chimeric constructs A-F and the nonhelical CT from bIFN-γ were based on hIFN-γ with the respective regions substituted with the corresponding bIFN-γ sequence. The sequence shown does not include the signal peptide. The alignment was made using Clustal W (www.ch.embnet.org/software/ClustalW.html; Larkin and others 2007). Symbols represent amino acids with identical (*), similar (:), or partly similar (.) side chains; below highly different amino acids, no symbol is shown. Amino acids shown by X-ray crystallography to interact with IFNGR1 are underlined (Thiel and others 2000) as is the KRKRS motif in CT implicated in receptor interaction (Döbeli and others 1988). IFN, interferon.

Mature monomeric hIFN-γ is 143 amino acids long with 2 N-linked glycosylation sites at positions 25 and 97; the fully glycosylated protein is the predominant form. Under physiological conditions, 2 IFN-γ chains self-associate noncovalently to a homodimer. The dimeric nature of the protein has been confirmed by X-ray crystallography showing that IFN-γ is primarily helical, with each monomer consisting of 6 alpha-helices (A-F) connected by short loops (Fig. 1A; Ealick and others 1991; Walter and others 1995). The dimer is formed when the C-terminal helices (E and F) from one chain associate head-to-tail with the N-terminal helices A, B, C, and D from the other chain.

The IFN-γ receptor is expressed on most cells and is composed of 2 chains. Following binding of IFN-γ, the high affinity subunit IFN-γ receptor alpha chain 1 (IFNGR1) interacts with the smaller subunit IFNGR2, which is required for IFN-γ signaling (Bach and others 1997). Signaling occurs via Janus kinase 1 (Jak1), Jak2, and signal transducer and activator of transcription 1 (STAT1) that after phosphorylation forms homodimers, which translocate inside the nucleus and initiate gene transcription (Bach and others 1997). The helical IFN-γ regions A and B and their connecting loop and helix F interact with the IFN-γ receptor (Lundell and Narula 1994; Thiel and others 2000).

An evolutionary conserved part of the C terminus (CT) has also been implicated in the receptor interaction but this has not been confirmed by X-ray crystallography due to the flexible nature of the CT (Lundell and Narula 1994).

The ability of antibodies to prevent cytokine-mediated receptor signaling depends on their specificity and epitope mapping is an important part of the characterization of neutralizing antibodies. MAbs to globular proteins generally recognize discontinuous, conformationally dependent epitopes that can be difficult to identify using peptides or protein fragments (Berzofsky and others 1982; Al Moudallal and others 1985; Meloen and others 1991). Instead, full-length proteins may have to be used for epitope mapping. Epitope mapping by X-ray crystallography is laborious and requires large amounts of pure protein and high quality crystals of antibody–antigen complexes.

Other strategies like expressing recombinant full-length proteins with point mutations, by site-directed mutagenesis or by use of variable display libraries, are used but may not result in structurally permissive substitutions; mutations affecting antibody binding may thus be a result of conformational changes rather than defining the amino acid residues involved in an epitope. A strategy decreasing that risk is to create chimeric proteins where regions are substituted by the corresponding region from a structurally homologous protein with a partially different amino acid sequence and that is not recognized by the mAbs being investigated (Lekcharoensuk and others 2004; Selga and others 2004; Cauwenberghs and others 2001). The probability that such substitutions introduced in the hybrid protein are structurally permissive is likely to be better compared to the use of random substitutions.

In this study, 12 mAbs against human IFN-γ were epitope mapped and evaluated for their capacity to prevent IFN-γ-signaling via its cellular receptor. Epitope mapping was performed using 7 different chimeric human-bovine IFN-γ constructs tagged with a peptide motif recognized by a specific mAb. The tag enabled quantification of the chimeras without purification and also facilitated a straightforward analysis of the mAbs' ability to bind the different chimeras. Expression of chimeras was made in HEK cells to facilitate optimal folding conditions and glycosylation.

## Materials and Methods

### Monoclonal antibodies to human IFN-*γ*

BALB/c mice and F344/Sca Fischer rats were immunized with recombinant *Escherichia coli*-derived hIFN-γ (Peprotech, Rockville Hill, NJ) in Abisco-100 adjuvant (Isconova, Uppsala, Sweden) and spleen cells were fused with Sp2/0 cells. Hybridomas were cultured and supernatants were analyzed by ELISA for reactivity with hIFN-γ. Positive hybridomas were subcloned and mAbs were purified from supernatants on Protein G columns. A portion of each purified mAb was biotinylated. For details on the above methods see Zuber and others 2005. Animals were housed at the Karolinska Institute, Solna, Sweden, and handled according to the guidelines of the Swedish Ethical Committee for Animal Protection. New mAbs made in this study were MT126L, 30S, 111W, 42H, 40K, 45F, 124i, and 11i. Previously made mAbs to IFN-γ included were 1-D1K, 7-B6-1, GZ4, and G23 (Mabtech, Nacka Strand, Sweden). All mAbs and their IgG subclasses are listed in Table 1.

**Table 1.** Monoclonal Antibodies to Human Interferon-γ and Their Relative Binding Strength

			*Affinity constant (KD)*
*Epitope cluster*^a^	*Clone name*	*Species and IgG subclass*	*(M)*^b^
A	GZ4	Mouse IgG1	1.6 × 10^−9^
A	1-D1K	Mouse IgG1	1.1 × 10^−9^
A	MT126L	Rat IgG2a	1.3 × 10^−9^
A	45F	Mouse IgG1	2.4 × 10^−9^
A	30S	Mouse IgG1	1.5 × 10^−9^
E	111W	Rat IgG2a	1.7 × 10^−9^
E	42H	Mouse IgG1	2.0 × 10^−9^
E	40K	Mouse IgG1	2.1 × 10^−9^
E	7-B6-1	Mouse IgG1	2.0 × 10^−9^
E	124i	Rat IgG1	2.4 × 10^−9^
Other	G23	Mouse IgG1	4.5 × 10^−9^
Other	11i	Mouse IgG1	3.0 × 10^−9^

^a^ For clarity, the epitope clusters that the mAbs later were defined to, are indicated.

^b^ The affinity constant (KD) at steady state was obtained by coating ELISA plates with mAbs followed by measurement of their capacity to bind biotinylated hIFN-γ. The KD value was calculated by dividing half of the maximal absorbance value (IC50) with the Mw of hIFN-γ.

### Recombinant IFN-*γ* and chimeras used for analysis of mAb specificity

Recombinant hIFN-γ and bovine (b) IFN-γ were produced based on sequences obtained from Uniprot (P01579 and P07353, respectively; Fig. 1B). The signal peptide from mouse IgG kappa (METDTLLLWVLLLWVPGSTGD) was included to enable secretion. Genes were codon optimized, synthesized, and cloned into the pIRES2-AcGFP1 plasmid (Clontech, Mountain view, CA) by GenScript (Piscataway, NJ). Seven human-bovine chimeric proteins were designed by replacing helical regions A-F or the C terminus (CT) of hIFN-γ with the corresponding residues from bIFN-γ (Fig. 1A); the substituted residues were 1–18 (chimera A), 19–36 (B), 37–62 (C), 63–82 (D), 83–98 (E), 99–121 (F), and 122–143 (CT). At the N terminus of all IFN-γ variants, a 10 amino acid tag (DAEFRHDSGY; designated BAM) was recombinantly added. The BAM tag is recognized by mAb bm-AbetaN (Mabtech). Proteins were expressed in transfected human HEK cells as previously described (Areström and others 2012). The transfection efficiency was estimated by analyzing mean fluorescence intensity of GFP expressed intracellularly using a Guava EasyCyte Mini flow cytometer (Merck Millipore, Billerica, MA). *E. coli*-derived hIFN-γ was biotinylated as described previously (Zuber and others 2005).

### Sandwich ELISA

Maxisorp 96-well plates (Nunc, Roskilde, Denmark) were coated (100 μL/well) for 16 h at 4°C with mAbs to IFN-γ diluted to 2 μg/mL in PBS. Other assay steps were at room temperature (RT), using 100 μL/well. Five washes using PBS with 0.1% Tween 20 were made between assay steps. After coating, wells were blocked for 1 h with incubation buffer (PBS with 0.05% Tween 20 and 0.1% bovine serum albumin). *E. coli*-derived hIFN-γ was diluted in incubation buffer to a concentration of 100 or 1,000 pg/mL and incubated for 2 h. After that, biotinylated detection mAb, diluted in incubation buffer to 1 μg/mL, was incubated for 1 h and subsequently streptavidin- horseradish peroxidase conjugate (SA-HRP; Mabtech) in incubation buffer was added and incubated for 1 h. The assay was developed with 3,3′,5,5′-tetramethylbenzidine substrate (Mabtech) and stopped with 1 M H_2_SO_4_ followed by absorbance measurement (450 nm) in an ELISA reader (Labsystems, Helsinki, Finland).

### Competitive ELISA

To assess whether mAbs competed for simultaneous binding to IFN-γ, each mAb was used as a capture mAb and evaluated for binding to biotinylated *E. coli*-derived hIFN-γ in the presence of competitive mAbs. Volumes, incubation times, buffers, and washes were the same as described above for the sandwich ELISA. First, to establish a suitable concentration of biotinylated hIFN-γ to use for each capture mAb, mAbs (2 μg/mL) were coated and incubated with a serial dilution of hIFN-γ-biotin followed by detection with SA-HRP. The concentration of hIFN-γ-biotin yielding 50% of the maximal absorbance value (IC50) for each mAb was later used in the competition ELISA; the IC50 value divided by the molecular weight of hIFN-γ also yields the affinity constant (KD) at steady state for the mAbs (Table 1). For the competitive ELISA, hIFN-γ -biotin, at the defined concentrations, was preincubated for 30 min with the mAbs to be tested for competition (0.2 μg/mL) before being added to ELISA plates coated with capture mAb. After incubation, bound IFN-γ-biotin was detected using SA-HRP. The percentage inhibition achieved by a competitor mAb was calculated by comparing absorbance values to hIFN-γ-biotin incubated in the absence of competitor mAb.

### Epitope mapping with human-bovine IFN-*γ* chimeras by ELISA

Epitope mapping of the individual mAbs was performed using the human/bovine IFN-γ chimeras N-terminally tagged with the BAM peptide. Using the sandwich ELISA protocol above, all mAbs in the panel were used as capture mAbs and incubated with serial dilutions of chimera supernatant of unknown concentration. Following that, bound chimeras were detected using biotinylated anti-BAM mAb. By comparing the relative binding of different mAbs to chimeras versus BAM-tagged wild-type (WT) hIFN-γ, it was observed that certain mAbs only displayed a loss of binding to a single chimera and others with multiple chimeras. For example, mAb 1-D1K only lost binding with chimera A and 7-B6-1 with chimera E. The recombinant WT hIFN-γ-BAM was then quantified using an established hIFN-γ ELISA system (Mabtech) based on mAb 1-D1K for capture and 7-B6-1-biotin for detection and with *E. coli*-derived hIFN-γ as a standard. The concentration of chimeras was then determined by comparing the IC50 value of the ELISA curve for each chimera with that of the WT hIFN-γ. Data obtained with 1-D1K/anti-BAM-biotin were used to quantify all chimeras except A and, similarly, 7-B6-1/anti-BAM was used to quantify all chimeras except E. For chimera concentrations determined by both mAbs, the average value was used. After having determined the concentration of chimeras and WT hIFN-γ in each HEK transfectant supernatant, the individual binding of coated mAbs was again determined using serial dilutions of WT hIFN-γ and chimeras with known concentration. WT bIFN-γ was included as a negative control. Percent binding of different mAbs to various chimeras was then compared to binding to WT hIFN-γ that was set to 100%.

### Biological activity of chimeras and IFN-*γ* neutralization assay using transfected HEK cells

HEK-Blue™ IFN-y cells (hIFN-γ sensor cells; InvivoGen, San Diego, CA) were cultured in DMEM supplemented with 10% FBS, 4.5 g/mL glucose, 50 U/mL penicillin, 50 μg/mL streptomycin, 100 μg/mL normocin, and 2 mM L-glutamine. Thirty μg/mL Blasticidin and 100 μg/mL Zeocin were used as selective antibiotics but were excluded when performing the experiments. Cells were maintained according to manufacturer's instructions. IFN-γ-mediated activation was assessed by incubating cells with hIFN-γ for 20 h whereafter 20 μL cell supernatant was collected and added to 200 μL Quanti-Blue™ substrate (InvivoGen) followed by analysis in an ELISA reader at 650 nm. For activation studies with chimeras, the human-bovine chimeras were serially diluted 1:5 starting at 10 ng/mL and added to 50,000 cells/well in a 96-well plate. Before performing neutralization experiments, the lowest *E. coli*-derived hIFN-γ concentration yielding a maximal signal was determined to be 100 pg/mL. To determine the neutralizing ability of mAbs, 100 pg/mL hIFN-γ was premixed with serial dilutions of the mAbs investigated (starting at 20 μg/mL) or isotype control mAbs. This mix was incubated for 60 min and then added to 50,000 cells/well for 20 h. Cell supernatants were collected and analyzed as above.

### Biological activity of chimeras and IFN-*γ* neutralization assay using primary human endothelial cells

Primary human aortic endothelial cells (HAEC; ATCC, Teddington, England) were cultured according to supplier's instructions in Vascular cell basal medium supplemented with Endothelial cell growth kit-VEGF (ATCC). HAEC were used for experiments in ≤8 passages and were cultured in 6-well plates. The total assay volume was 1.5–2 mL. For assessing the biological activity of chimeras, HAEC were stimulated with 0.5 and 5 ng/mL of each chimera (A-F and CT) for 48 h. WT hIFN-γ and bIFN-γ were used as positive and negative controls, respectively, at the same concentrations. HAEC were subsequently detached using 1 mM EDTA/PBS and stained for MCH class II expression (0.1 μg/test of mAb HB55; ATCC) for 25 min at +4°C. Following washing, the MHC class II staining was revealed using PE-conjugated F(ab')_2_ goat anti-mouse IgG for 25 min at +4°C in the dark (diluted 1:50; Jackson ImmunoResearch, Inc., West Grove, PA). The MHC class II expression was detected by flow cytometry using a Guava EasyCyte Mini (Merck Millipore) and data were analyzed in Flow Jo software (Flow Jo LLC, Ashland, CA). Ten thousand events were acquired for each sample. To investigate the neutralization efficiency of mAbs 1-D1K, 30S, 111W, and 124i on HAEC activation by IFN-γ, HAEC were incubated with 1 ng/mL *E. coli*-derived recombinant hIFN-γ mixed with 0.001, 0.01, and 0.1 μg/mL of the respective mAbs or matched isotype control Abs for 48 h. One ng/mL of IFN-γ was used since this was the lowest hIFN-γ concentration yielding maximal activation in the absence of any mAbs. Flow cytometry was then performed as described above.

## Results

Twelve mAbs to hIFN-γ (Table 1) were analyzed to relate their epitope specificity to their ability to neutralize IFN-γ. Assessment of the mAbs' ability to capture biotinylated hIFN-γ showed a comparable affinity for the mAbs with KD values around 1–2 × 10^−9^ except for mAb G23 and 11i that displayed somewhat weaker binding (Table 1).

### Epitope clusters defined by sandwich ELISA

**FIG. 2.**
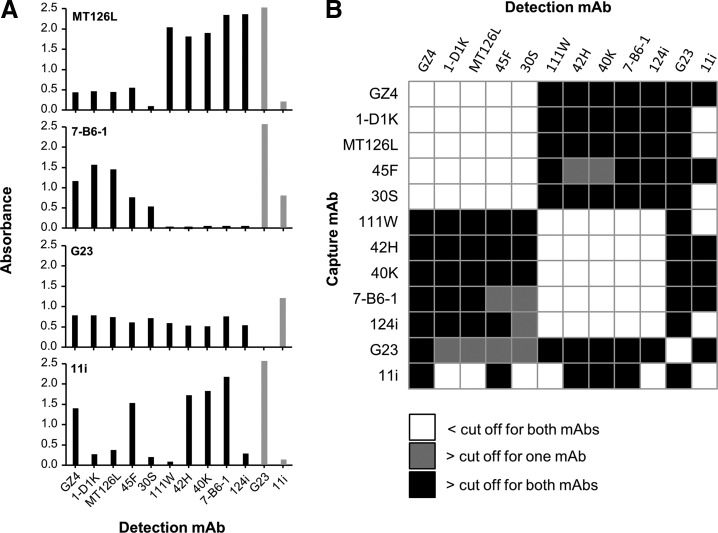
Epitope mapping by sandwich ELISA. All mAbs were analyzed in all possible combinations as capture and detection mAbs for reactivity with hIFN-γ in ELISA. **(A)** Example graphs are shown for mAbs MT126L, 7-B6-1, G23, and 11i used for capture in combination with each mAb in the panel as detection mAb. The analysis was made with 100 pg/mL of hIFN-γ and the background (0 pg/mL) was subtracted. When G23 and 11i was used as detection mAbs, 1,000 pg/mL of hIFN-γ was used since these mAbs yielded poor signals with 100 pg/mL when used as detection mAbs (indicated by *gray bars*). **(B)** The graph summarizing the functionality of mAb combinations is based on a cutoff definition for a positive signal. A signal was defined as positive for both mAbs (*black boxes*) if the absorbance value was >0.5 and if the signal was 2.5× higher than the signal obtained when using either of the mAbs in a homologous combination (i.e., the same mAb used for capture as well as detection). If only one mAb fulfilled the criteria the result is shown as >cutoff for one mAb (*gray boxes*). Combination of mAbs where none of the mAbs fulfilled the criteria are shown as <cutoff for both mAbs (*white boxes*). Data shown are the mean of 2 experiments.

When analyzed by sandwich ELISA, the mAbs clustered into 2 major groups (denoted the MT126L and the 7-B6-1 group, respectively) where antibodies in each group could bind to IFN-γ simultaneously and without interference from each other, indicating recognition of distinct epitope regions (Fig. 2). Due to the homodimeric nature of IFN-γ, combinations of capture and detection antibodies from the same epitope group, including the use of the same antibody in both positions, also gave a certain signal in the ELISA although much lower compared to when using antibodies from the separate clusters. The functionality of mAb MT126L and 7-B6-1 as capture mAbs with other detection mAbs is shown in detail in Figure 2 and is representative for the respective epitope groups.

Two mAbs (G23 and 11i) did not cluster in the major groups (Fig. 2). MAb G23 was functional with all other mAbs and thus recognized a unique epitope. MAb 11i, on the other hand, was functional with G23 and with 2 out of 5 mAbs from each cluster, demonstrating that the 11i epitope overlaps with both major epitope regions but also that the mAbs within each cluster recognize overlapping but not identical epitopes.

### Epitope clusters defined by competitive ELISA

**FIG. 3.**
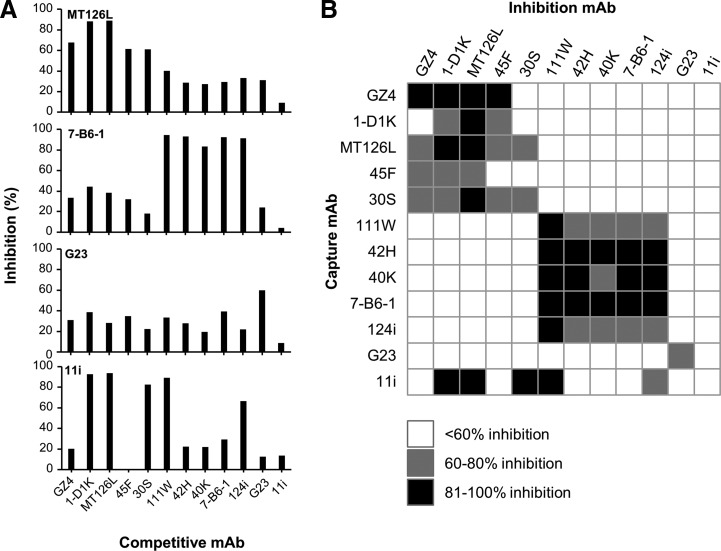
Epitope mapping by competitive ELISA. Each mAb, used as a capture mAb, was allowed to bind biotinylated hIFN-γ that had been premixed with 0.2 μg/mL of a competitive mAb. The concentration of hIFN-γ used for each capture mAb was determined by their IC50 value (half the maximal absorbance value) when mAbs were allowed to bind a serial dilution of hIFN-γ in the absence of any competitive mAb (Table 1). **(A)** The capacity of a competitive mAb to inhibit the binding of hIFN-γ by the capture mAb is shown in graphs for MT126L, 7-B6-1, G23, and 11i. **(B)** The graph summarizing the competitive ELISA data shows the inhibition for each combination of capture and competitive mAb as 100%–81% (*black boxes*), 80%–60% (*gray boxes*), and <60% (*white boxes*). Data shown are the mean of 2 experiments.

Overall, the competitive ELISA corroborated the results of the sandwich ELISA (Fig. 3) but since the competitive ELISA is affected by the respective affinity of the 2 mAbs competing for binding, some mAbs failed to inhibit other mAbs expected to bind an overlapping epitope. MAb 11i, for example, shown to bind IFN-γ weaker than most other mAbs (Table 1), was inhibited by mAbs shown to bind overlapping epitopes in sandwich ELISA but did not itself inhibit any mAb. The results shown are based on using 0.2 μg/mL of inhibition mAb. When increasing the concentration of the competitive mAb to 20 μg/mL, inhibition was also observed with other combinations of mAbs where inhibition could be expected. For example, mAb 11i could inhibit itself and mAb 30S. Also mAb 45F could be inhibited by itself and also by mAb 30S. At a higher concentration, mAb 45F, in contrast to all other mAbs in the MT126L group, also displayed inhibition of all mAbs defined by sandwich ELISA to belong to the other epitope cluster that is, the 7-B6-1 group.

### Identification of epitope regions using chimeric human-bovine IFN-*γ* constructs

**FIG. 4.**
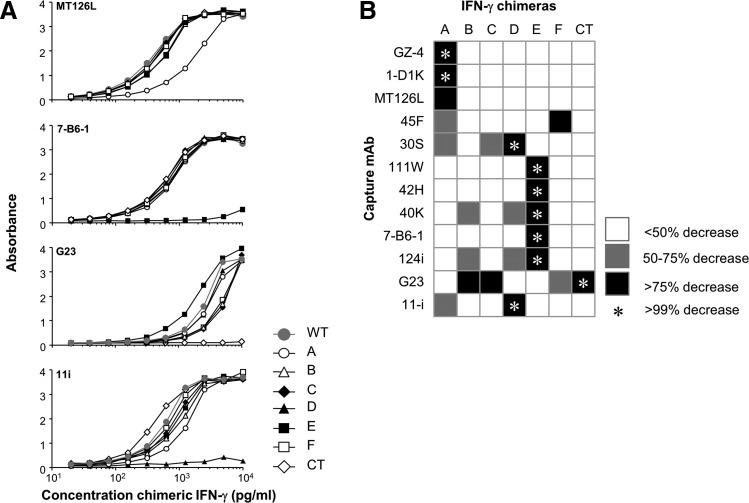
Identification of epitope regions using chimeric human-bovine IFN-γ. Seven hIFN-γ constructs, each with one helical region (A–F) or the C terminus (CT) substituted with the corresponding bIFN-γ region, were compared with WT hIFN-γ for binding to all mAbs. MAbs were used as capture mAbs and allowed to bind serial dilutions of the chimeras and WT hIFN-γ followed by detection of bound protein using an anti-tag mAb. **(A)** Example graphs are shown for MT126L, 7-B6-1, G23 and 11i. bIFN-γ was not recognized by any mAb (not shown). **(B)** Summary of the chimeras causing a decreased binding by the respective mAbs. The loss of binding is shown as a complete loss (*), >75% (*black boxes*), 50%–75% (*gray boxes*), and <50% (*white boxes*). The percentual loss of binding was calculated by comparing the concentration of each chimera yielding half the maximal absorbance value against the concentration of WT IFN-γ yielding half the maximal absorbance value. Data shown are the mean of 2 experiments.

The mAbs were initially tested in western blot for reactivity with hIFN-γ. Only mAb 111W yielded a strong signal whereas mAbs 7-B6-1 and G23 reacted weakly and the other mAbs did not work, suggesting a predominant recognition of conformationally dependent epitopes (data not shown). To identify the actual location of the epitopes on the surface of hIFN-γ, each helical region and their connecting loop regions in hIFN-γ and the CT were replaced with the corresponding residues from bIFN-γ (Fig. 1B) and the mAb binding to the chimeras was assessed. Each mAb displayed a complete or partial loss of binding with one or several chimeras (Fig. 4 and Table 2). MAbs in the MT126L group lost binding when helix A was substituted although the binding of 2 mAbs (45F and 30S) were more impaired by substitutions in region D or F. All mAbs in the 7-B6-1 group completely failed to bind the E chimera and 2 of them (40K and 124i) also showed a decrease in binding with chimera B and D.

**Table 2.** Epitope Specificity and Neutralizing Capacity of Monoclonal Antibodies to Human Interferon-γ

			*Neutralization*
*Epitope cluster*	*Clone name*	*Epitope determinants*^a^	*(ND50; pM)*^b^
A	GZ4	A	3500
A	1-D1K	A	390
A	MT126L	A	>13000
A	45F	a,F	1400
A	30S	a,c,D	—
E	111W	E	40
E	42H	E	53
E	40K	b,d,E	390
E	7-B6-1	E	120
E	124i	b,d,E	9100
Other	G23	B,C,f,CT	—
Other	11i	a,D	—

^a^ The letters indicate the loss of mAb reactivity obtained with the chimeras (Fig. 4) with upper case letters representing a >75% loss of reactivity and lower case letters representing 50%–75% loss of reactivity.

^b^ ND50 represents the concentration of mAb required to decrease IFN-γ-mediated (5.95 pM) activation of HEK cells with 50%. Data are the mean of 3 experiments.

MAb 11i, recognizing an epitope overlapping with both major epitope regions, lost binding when region D was replaced and partially when region A was replaced. G23, with a unique epitope, was primarily affected by substitutions in CT but also partially by substitutions in B, C, and F.

### Activation of IFN-*γ*-responsive cells using human-bovine IFN-*γ* chimeras

**FIG. 5.**
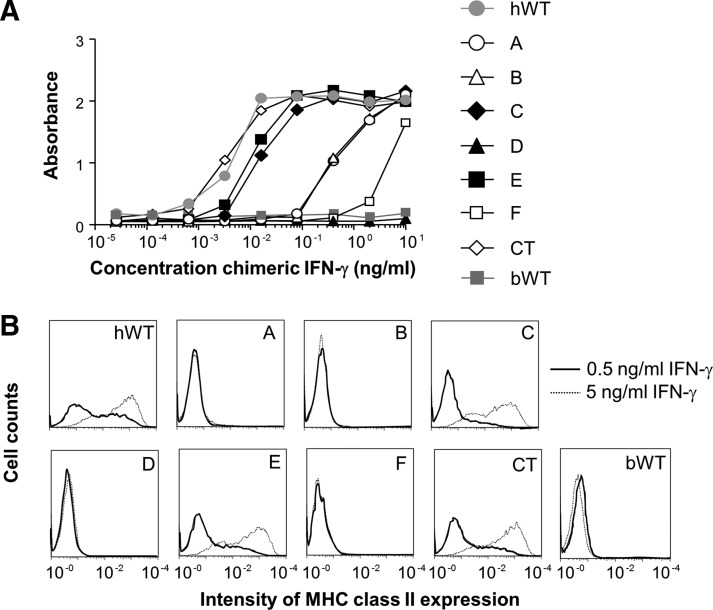
IFN-γ-mediated activation of human cells with human or bovine wild-type IFN-γ or human-bovine IFN-γ chimeras. **(A)** HEK *BLUE*™ cells were incubated with serial dilutions of human IFN-γ WT (hWT), human-bovine IFN-γ chimeras, or bovine IFN-γ WT (bWT). After 20 h incubation, ALP secreted into the supernatant in response to IFN-γ activation was measured in an ELISA reader at 650 nm. bIFN-γ did not activate the cells. **(B)** Human aortic endothelial cells, responding to hIFN-γ by expression of MHC class II, were incubated with 0.5 and 5 ng/mL of hWT, human-bovine IFN-γ chimeras or bWT. After 48 h incubation, MHC class II expression was analyzed by flow cytometry and 10,000 events were acquired. bIFN-γ did not activate the cells.

To assess whether the chimeras displayed a preserved capacity to interact with the IFN-γ receptor, the chimeras were compared with WT hIFN-γ for their ability to activate HEK Blue™ cells responsive to hIFN-γ (Fig. 5A). Chimera C, E, and CT displayed an activating capacity comparable to WT. The 3 chimeras A, B and F, together forming the suggested active site interacting with the IFN-γ receptor, all resulted in a partial loss of activation. Notably, substitution of region D, not described to be involved in the receptor interaction, resulted in a complete loss of activation. The results were confirmed using primary human endothelial cells that, after stimulation with IFN-γ, increase their expression of MHC class II. These cells responded in a comparable manner to the WT, C, E, and CT chimeras, whereas chimeras A, B, D, and F all failed to activate the cells (Fig. 5B).

### MAb neutralization of IFN-*γ*-mediated receptor signaling

**FIG. 6.**
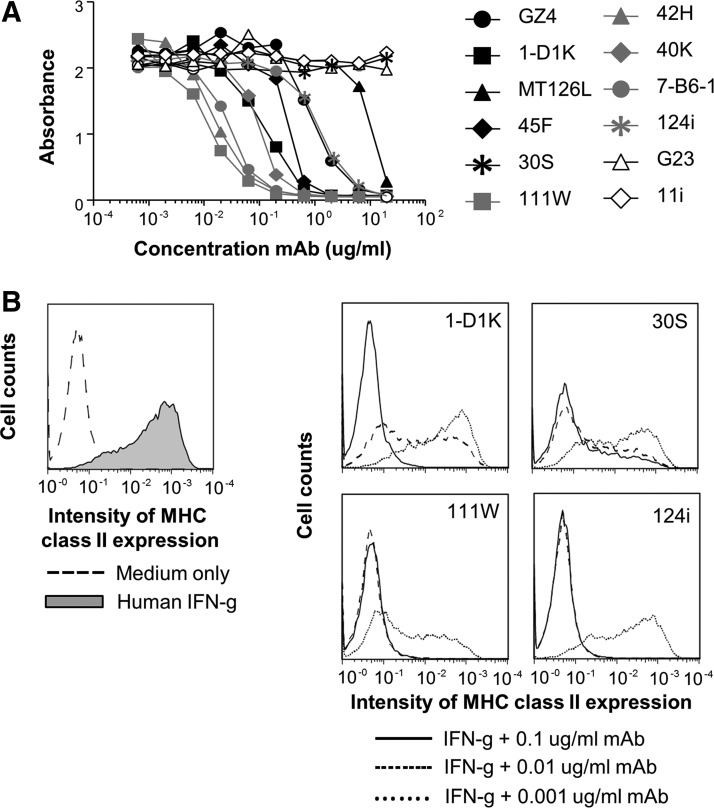
Ability of mAbs to neutralize IFN-γ-mediated activation of human cells. **(A)** HEK *BLUE*™ cells were incubated with 100 pg/mL hIFN-γ, with or without serial dilutions of mAbs to hIFN-γ. IFN-γ-induced enzyme secretion was measured after 20 h. The addition of anti-IFN-γ mAbs in the absence of hIFN-γ had no effect on the cells (not shown). Addition of isotype control mAbs to IFN-γ-activated cells had no neutralizing effect (not shown). Data shown are one of 3 representative experiments. **(B)** Neutralization of IFN-γ-induced activation of human aortic endothelial cells. Endothelial cells were incubated with 1 ng/mL of recombinant hIFN-γ, with or without serial dilutions of selected mAbs to hIFN-γ. IFN-γ-mediated MHC class II expression was analyzed by flow cytometry after 48 h. The graph to the *left* shows unstimulated cells (*hatched line*) and IFN-γ-stimulated cells without neutralizing mAb (*solid line*, *gray* field). The graphs to the *right* show neutralization with 4 different mAbs at 3 concentrations. The addition of anti-IFN-γ mAbs in the absence of hIFN-γ had no effect on the cells (not shown). Addition of isotype control mAbs to IFN-γ-activated cells had no neutralizing effect (not shown). Data shown are one of 2 experiments with reproducible results.

The mAbs were further analyzed for their neutralizing capacity as defined by their inhibition of hIFN-γ-mediated signaling via its receptor on HEK Blue™ IFN-γ cells (Fig. 6A and Table 2). All mAbs recognizing region E were neutralizing with 4 mAbs having ND50 values of 0.04–0.39 nM, whereas one mAb (124i) was less efficient. In the A-reactive group, only mAb 1-D1K had an ND50 value comparable to the E group mAbs (0.39 nM). Three other mAbs in the A group had a neutralizing effect but weaker and one had no effect (30S). MAbs 11i and G23 had no neutralizing capacity.

The neutralizing capacity of the most and least efficient mAbs in the A and E cluster was confirmed using hIFN-γ-mediated activation of primary human endothelial cells and MHC class II expression as read-out (Fig. 6B). MAb 111W, the most efficient neutralizing mAb using HEK cells, was also the most efficient inhibitor of MHC class II expression. The least efficient mAb in the E group in the analysis with HEK cells, mAb 124i, was less efficient than 111W but more efficient than mAb 1-D1K in the A group. MAb 30 S from the A group, which had no neutralizing effect with HEK cells, displayed a neutralizing capacity with primary human cells but failed to completely block activation even at the highest mAb concentration.

Compared to primary HAEC cells, HEK cells required a lower minimal dose of hIFN-γ for maximal activation but a higher concentration of, for example, mAb 111W to achieve complete neutralization. This rather big difference between the assays with regard to the molar ratio of hIFN-γ levels required for activation and the level of inhibitory mAb required for complete neutralization is likely explained by the level of expression of receptor chains and/or other components of the signaling pathway by the different cells. HAEC express IFN-γ receptors and the signaling pathway molecules naturally, whereas HEK cells express IFN-γ receptors and certain signaling molecules naturally (although at potentially different levels than HAEC) but are transfected by plasmids encoding STAT1, which is likely to result in an overexpression of STAT1 and potentially a different responsiveness to IFN-γ.

## Discussion

Different strategies have been applied when using chimeras for epitope mapping and studies of receptor interactions. For both types of analyses, chimeras have been used without purification, either in supernatants or expressed on cells, with the limitation that results will not be quantifiable, only positive or negative (Thakur and Landolfi 1999; Lekcharoensuk and others 2004; Cauwenberghs and others 2001). To accomplish a quantitative comparison, chimeric proteins have, however, also been used in purified form (Lundell and others 1994; Selga and others 2004). The inclusion of a peptide tag in the human-bovine IFN-γ chimeras used herein facilitated a quantitative analysis of both epitope binding and the interaction between hIFN-γ and its receptor, without the need for purification.

Using the chimeric IFN-γ variants, where the helical regions A-F and their interconnecting loops, along with the CT region were replaced one by one with the respective bIFN-γ residues, the epitopes of 12 mAbs to hIFN-γ were identified. Ten of the 12 mAbs clustered in 2 distinct epitope groups (5 mAbs in each group) with the recognition of helical region A or E as their respective common denominator. All antibodies binding in the E region were neutralizing and able to efficiently block IFN-γ-induced activation, whereas mAbs binding to the A region were generally less potent and 2 lacked neutralizing capacity.

Previously, when a panel of 21 neutralizing mAbs to hIFN-γ was analyzed for simultaneous binding to hIFN-γ in a competitive radioimmunoassay, the mAbs could, in a similar manner, be clustered in 2 major epitope groups (Alfa and Jay 1988), one of which was later shown to involve helix E (Zu and Jay 1991). The location of the other major epitope region was not identified but, given the neutralizing capacity of the antibodies, it might well correspond to the helical A region recognized as one of the major epitope clusters in this study.

Similar to many mAbs raised to globular proteins (Berzofsky and others 1982; Al Moudallal and others 1985), most mAbs analyzed herein recognized conformational epitopes, as judged from their poor reactivity with hIFN-γ in western blot and, for several mAbs, recognition of multiple chimeras.

Initially the mAbs were also analyzed in western blot for reactivity with recombinant fragments of hIFN-γ each comprising 2–3 helical regions. MAb 111W, one of the mAbs in the E group and the only mAb yielding a strong signal with WT hIFN-γ in western blot, weakly recognized a fragment spanning helices C,D, and E whereas all other mAbs were nonreactive with all fragments (unpublished observations). This lack of reactivity is not surprising since fragments will not assemble into dimeric structures and also because the helical regions juxtapositioned in the IFN-γ dimers and potentially forming discontinuous epitopes, are not necessarily close in the primary structure.

In the case of certain epitopes, for example, for mAb 45F mapping to regions A and F, the 3-dimensional structure of IFN-γ suggests that the 2 parts of this discontinuous epitope are from different chains in the IFN-γ dimer. Although the mAbs analyzed in the present study, except for mAb 111W, required full-length hIFN-γ for efficient binding, Zu and Jay 1991 defined a major mAb cluster as specific for residue 84–94 in the E helix using an octamer peptide scan. However, similar to certain mAbs herein, which lost reactivity with several chimeras indicating that each chimera is likely to represent a different part of a discontinuous epitope, a short peptide may represent a linear epitope but can also be the major region of a discontinuous epitope (Meloen and others 1991; Cauwenberghs and others 2001).

An indication of a predominant recognition of discontinuous epitopes by mAbs to IFN-γ was also seen when a panel of anti-bIFN-γ mAbs was mapped with the same chimeras used herein (unpublished observations). In this reversed situation, from the perspective of the anti-bIFN-γ mAbs, the chimeras comprise only one helical region from bIFN-γ, within a hIFN-γ backbone (although with an overall 62% identity). Out of 19 mAbs reactive with WT bIFN-γ, only 3 mAbs were able to bind a chimera. Notably, all 3 mAbs recognized the chimera comprising the bIFN-γ E region.

Each chimera was recognized by at least half the panel of anti-hIFN-γ mAbs, demonstrating that the chimeras maintained their antigenicity and structurally resembled WT hIFN-γ. The preservation of a seemingly intact structure of the chimeras may relate to the fact that bIFN-γ is homologous enough to hIFN-γ to only have a subtle impact on the overall hIFN-γ structure, while still introducing side chain modifications affecting mAbs recognition. Also, the expression in human cells is likely to facilitate optimal folding and glycosylation. When murine-human IFN-γ chimeras were expressed in *E. coli* to study the interaction with the hIFN-γ receptor, chimeras of hIFN-γ with the helical region A, E, or F substituted with mouse residues were insoluble and could not be further analyzed (Lundell and others 1994).

Maintaining a preserved structure of the chimeras by avoiding introduction of too many modified residues comes at a price with regard to the epitope mapping. Only if the bIFN-γ residues introduced in the hIFN-γ backbone, by chance, replace amino acids important for the binding of a mAb will the binding be significantly reduced. It is thus possible that epitopes of certain mAbs, determined to be located within one helical region, involve other regions where crucial amino acids are unchanged or substituted with side chains that have little impact on the mAb binding. A finding suggesting that epitopes can be more complex than revealed in the chimera analysis was that mAb 45F, mapped to helical region A and F, had the ability to, at high concentrations, block the binding of mAbs mapped to the E region.

Also, the difference between a chimera resulting in a complete loss of mAb reactivity versus a chimera just causing a partial loss of reactivity may actually be due to which amino acids that are substituted rather than indicating which part of a discontinuous epitope that is most crucial. A finding that could support this reasoning is that the 2 chimeras with the lowest proportion of substituted residues (C = 19%; CT = 27%) together only affected the binding of 3 mAbs, whereas the chimeras with most substitutions (E = 62%; D = 50%) had an impact on the binding of 9 mAbs. Obviously, the bias for mAb recognition of certain regions is likely to also explain why these chimeras had more impact on the mAb binding.

A well-maintained structure of the chimeras was further suggested by the ability of chimera C, E, and CT to activate hIFN-γ receptor-expressing cells in a manner similar to WT IFN-γ. Chimera A, B, and F displayed a reduced activating capacity and amino acids in these regions (Fig. 1B) have previously been shown to bind the IFNGR1 chain by X-ray crystallography (Thiel and others 2000). Studies utilizing murine-human IFN-γ chimeras have also indicated the loop connecting the helical regions A and B as important for the receptor interaction (Lundell and others 1994) and a mutation of His_111_ to Asp_111_ in the F helix abolishes the mutant's activating capacity (Lunn and others 1992). All these 3 chimeras used here (A, B, and F), differ between hIFN-γ and bIFN-γ in several residues implicated in the receptor interaction, most notably the F chimera that has the bovine residue Asn_111_.

Also residues 128–132 (KRKRS) of the C terminus have been suggested to be involved in receptor interaction (Döbeli and others 1988) but this has not been confirmed in crystal structures due to the flexibility of the C terminus. These residues are completely conserved between human and cow and many other mammals, which may explain why the CT chimera retained full biological activity. Unexpectedly, chimera D completely abrogated receptor signaling despite the fact that it has not been described to be involved in the receptor interaction. The structure of chimera D should be relatively intact considering that 8 of the mAbs displayed reactivity comparable to their reactivity with WT hIFN-γ but a subtle difference in structure could have an impact on the receptor interaction without significant effects on the antigenicity.

In line with the involvement of helical region A in the binding to IFNGR1, several mAbs directed to this region inhibited hIFN-γ-mediated activation of human cells, likely due to a direct block of IFN-γ binding to its receptor. Still, 2 mAbs displayed no, or a poor, neutralizing capacity, which cannot be explained only by affinity as their binding strength to IFN-γ was comparable with other efficiently neutralizing mAbs. Thus, subtle differences in their specificity are likely to explain their different capacity to prevent the receptor interaction.

Notably, all mAbs recognizing the helical region E displayed a neutralizing capacity and several of them with a capacity that surpassed that of the A epitope group. MAbs reactive with the E region have been shown previously to be neutralizing and it has been suggested that the E region of hIFN-γ contains a stretch of amino acids (KKKRD) important for nuclear targeting (Alfa and Jay 1988; Zu and Jay 1991). However, this theory has been partly contradicted by experiments showing that these residues could be substituted with the corresponding bIFN-γ residues (SEKLE) with only a partial loss (30%–40%) of the biological activity (Lundell and Narula 1994); a finding that was corroborated by the results herein.

The mechanism for how mAbs to region E neutralize hIFN-γ is thus not known but an alternative mechanism for neutralization of IFN-γ has been described for the humanized mAb NI-0501, which was observed to bind both free and IFNGR1-bound IFN-γ (Hatterer and others 2012). Using an *in situ* proximity ligation assay, mAb NI-0501 was observed to impair IFNGR1 and IFNGR2 interaction induced by hIFN-γ at the cell surface. Although the epitope recognized by mAb NI-0501 has not been identified, the same mechanism may explain the efficient neutralization displayed by mAbs to region E.

The use of peptide-tagged chimeras facilitated a straightforward identification of conformational epitopes recognized by mAbs to IFN-γ. Exactly the same approach as used herein was recently used to successfully identify conformational epitopes recognized by neutralizing mAbs to human IL-21 (unpublished observations) and should be possible to use for the identification of epitopes recognized by neutralizing antibodies against a variety of cytokines or other proteins.
